# Overexpression of Nmnat3 efficiently increases NAD and NGD levels and ameliorates age‐associated insulin resistance

**DOI:** 10.1111/acel.12798

**Published:** 2018-06-14

**Authors:** Maryam Gulshan, Keisuke Yaku, Keisuke Okabe, Arshad Mahmood, Tsutomu Sasaki, Masashi Yamamoto, Keisuke Hikosaka, Isao Usui, Tadahiro Kitamura, Kazuyuki Tobe, Takashi Nakagawa

**Affiliations:** ^1^ Frontier Research Core for Life Sciences University of Toyama Toyama Japan; ^2^ Department of Metabolism and Nutrition Graduate School of Medicine and Pharmaceutical Science for Research University of Toyama Toyama Japan; ^3^ First Department of Internal Medicine Graduate School of Medicine and Pharmaceutical Science for Research University of Toyama Toyama Japan; ^4^ Laboratory of Metabolic Signal Metabolic Signal Research Center Institute for Molecular and Cellular Regulation Gunma University Maebashi Japan; ^5^ Department of Otorhinolaryngology‐Head and Neck Surgery Osaka University Graduate School of Medicine Osaka Japan; ^6^ Institute of Natural Medicine University of Toyama Toyama Japan

**Keywords:** aging, insulin resistance, NGD, nicotinamide adenine dinucleotide, Nmnat3, reactive oxygen species

## Abstract

Nicotinamide adenine dinucleotide (NAD) is an important cofactor that regulates various biological processes, including metabolism and gene expression. As a coenzyme, NAD controls mitochondrial respiration through enzymes of the tricarboxylic acid (TCA) cycle, β‐oxidation, and oxidative phosphorylation and also serves as a substrate for posttranslational protein modifications, such as deacetylation and ADP‐ribosylation by sirtuins and poly(ADP‐ribose) polymerase (PARP), respectively. Many studies have demonstrated that NAD levels decrease with aging and that these declines cause various aging‐associated diseases. In contrast, activation of NAD metabolism prevents declines in NAD levels during aging. In particular, dietary supplementation with NAD precursors has been associated with protection against age‐associated insulin resistance. However, it remains unclear which NAD synthesis pathway is important and/or efficient at increasing NAD levels in vivo. In this study, Nmnat3 overexpression in mice efficiently increased NAD levels in various tissues and prevented aging‐related declines in NAD levels. We also demonstrated that Nmnat3‐overexpressing (Nmnat3 Tg) mice were protected against diet‐induced and aging‐associated insulin resistance. Moreover, in skeletal muscles of Nmnat3 Tg mice, TCA cycle activity was significantly enhanced, and the energy source for oxidative phosphorylation was shifted toward fatty acid oxidation. Furthermore, reactive oxygen species (ROS) generation was significantly suppressed in aged Nmnat3 Tg mice. Interestingly, we also found that concentrations of the NAD analog nicotinamide guanine dinucleotide (NGD) were dramatically increased in Nmnat3 Tg mice. These results suggest that Nmnat3 overexpression improves metabolic health and that Nmnat3 is an attractive therapeutic target for metabolic disorders that are caused by aging.

## 1 INTRODUCTION

As a coenzyme, nicotinamide adenine dinucleotide (NAD) regulates various metabolic enzymes through redox reactions. More than half of cellular NAD resides in the mitochondria, and the activities of NAD‐dependent enzymes within the tricarboxylic acid (TCA) cycle, β‐oxidation, and oxidative phosphorylation are directly influenced by NAD concentrations (Stein & Imai, 2012). Mitochondria efficiently produce ATP through aerobic respiration but are also the principal source of reactive oxygen species (ROS), which are considered the central cause of aging (Bratic & Larsson, 2013). Dysfunction of mitochondria increases the production of ROS and promotes DNA damage in cells and tissues, ultimately contributing to aging. Thus, NAD is considered a critical determinant of ATP and ROS production in the mitochondria (Murphy, 2009). Furthermore, NAD is a substrate for poly(ADP‐ribose) polymerase (PARP) and sirtuins, which catalyze ADP‐ribosylation and deacetylation, respectively (Canto, Menzies, & Auwerx, 2015; Li et al., 2017). Sirtuins are well‐known antiaging molecules with diverse biological functions in metabolism, gene expression, and cellular stress responses. Thus, NAD is considered a nexus for metabolism and aging. Numbers of studies show that NAD levels decrease with aging in multiple organs and that declines in intracellular NAD concentrations are related to various aging‐associated phenotypes (Canto et al., 2015; Rajman, Chwalek, & Sinclair, 2018; Yoshino, Baur, & Imai, 2017). Therefore, increases in intracellular NAD levels may combat with aging‐associated diseases. Accordingly, several studies have demonstrated that dietary supplementation with NAD precursors, such as nicotinamide mononucleotide (NMN) and nicotinamide riboside (NR), efficiently increases NAD levels in certain tissues and prevents aging‐ and diet‐associated obesity and insulin resistance in mice (Canto et al., 2012; Yoshino, Mills, Yoon, & Imai, 2011).

In mammalian cells, NAD is predominantly synthesized through the salvage pathway, for which nicotinamide phosphoribosyltransferase (Nampt) is a rate‐limiting enzyme (Revollo, Grimm, & Imai, 2004). Nampt catalyzes the formation of NMN from nicotinamide (NAM) and 5‐phosphoribosyl‐pyrophosphate (PRPP), and NMN is subsequently converted to NAD by nicotinamide mononucleotide adenylyltransferase (Nmnat; Berger, Lau, Dahlmann, & Ziegler, 2005; Nikiforov, Dolle, Niere, & Ziegler, 2011). However, it remains unclear which NAD synthesis enzyme can be exploited to boost NAD levels in vivo. Because mitochondrial NAD regulates various metabolic pathways through redox enzymes and mitochondrial sirtuins, increased mitochondrial NAD levels may ameliorate various aging‐associated phenotypes, including insulin resistance (Gomes et al., 2013; Zhang et al., 2016). Originally, Nampt was considered a cytoplasmic protein; however, it has been found in the nuclei and mitochondria (Yang et al., 2007). Previously, to investigate the effects of increased NAD biosynthesis in vivo, muscle‐specific Nampt‐overexpressing mice were generated (Frederick et al., 2015). Although NAD levels in skeletal muscle were significantly elevated, mitochondrial oxidative phosphorylation was not promoted in these mice. Thus, we investigated Nmnat as a target enzyme that may increase mitochondrial NAD levels. In mammals, there are three Nmnat isozymes encoded by different genes, namely, Nmnat1, Nmnat2, and Nmnat3 (Berger et al., 2005). In particular, Nmnat3 has been found in the mitochondria and is widely associated with mitochondrial NAD biosynthesis (Berger et al., 2005; Nikiforov et al., 2011; VanLinden et al., 2015). Although Nmnat3 deficiencies in mice caused no obvious changes in mitochondrial NAD levels, suggesting redundancy between mammalian Nmnat isozymes (Hikosaka et al., 2014; Yamamoto et al., 2016), overexpression of Nmnat3 in human cultured cells reportedly contributed to mitochondrial NAD synthesis (Nikiforov et al., 2011). These data suggest that overexpression of Nmnat3 can increase mitochondrial NAD levels and stimulate mitochondrial metabolism in vivo. In addition, Nmnat3 has been shown to generate the NAD analogs nicotinamide guanine dinucleotide (NGD) and nicotinamide hypoxanthine dinucleotide (NHD) in vitro. However, the presence of these NAD analogs has not been verified in vivo, and the ensuing biological functions are completely unknown (Berger et al., 2005). In this study, we employed Nmnat3‐overexpressing (Nmnat3 Tg) mice and assessed the impacts of Nmnat3 gain of function on NAD metabolism and aging‐associated phenotypes, including insulin resistance. Taken together, the present data show novel beneficial effects of Nmnat3 against aging.

## 2 RESULTS

### 2.1 NAD levels are increased in various tissues of Nmnat3 Tg mice

**Figure 1:**
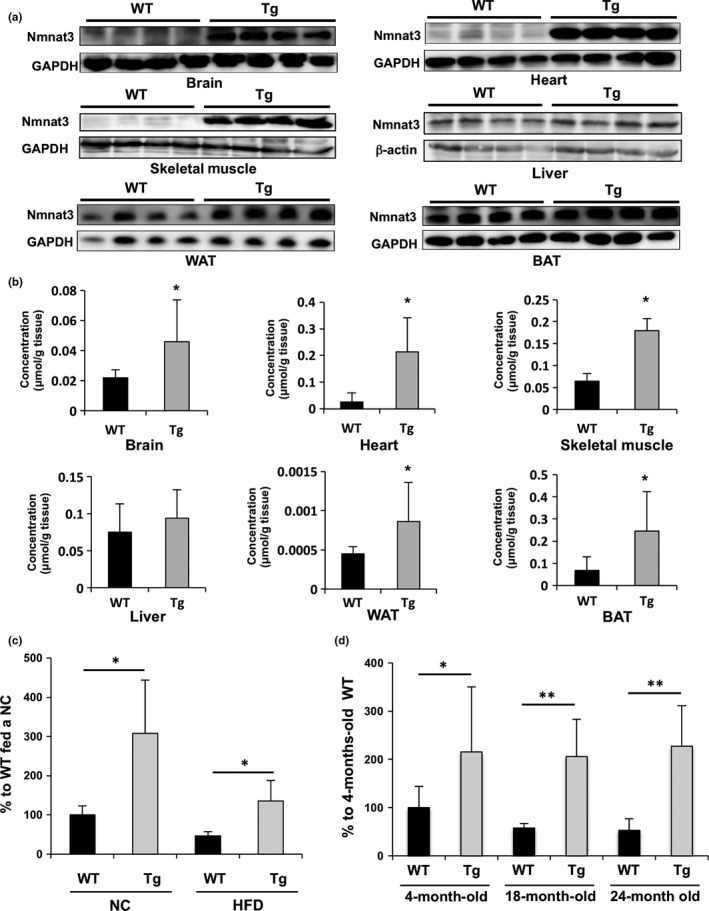
Nmnat3 Tg mice maintained the NAD levels even after the HFD feeding and aging. (a) Immunoblot analysis of Nmnat3 expression in liver, skeletal muscle, heart, and WAT (white adipose tissue), BAT (brown adipose tissue), and brain from 4‐month‐old Nmnat3 Tg and wild‐type (WT) mice (*n* = 4 for each group). GAPDH or β‐actin was used as loading control. (b) Absolute quantification of NAD levels by LC/MS. Tissue samples were prepared from 4‐month‐old WT and Nmnat3 Tg mice. Data are presented as mean ± *SD* (*n* = 8 for each group). (c) Semiquantification of NAD levels by LC/MS using tissue samples prepared from female WT and Nmnat3 Tg mice fed the NC or HFD for 10 weeks. Data are presented as mean ± *SD* (*n* = 8 for each group). (d) Semiquantification of NAD levels by LC/MS using issue samples prepared from female WT and Nmnat3 Tg mice at the ages of 4, 18, and 24 months. Data are presented as mean ± *SD* (*n* = 8 for each group). Single (*) and double (**) asterisk indicate that *p*‐value was <0.05 and 0.01, respectively

To elucidate the benefits of increased Nmnat3 expression in vivo, we employed Nmnat3‐overexpressing (Nmnat3 Tg) mice in which Nmnat3 was ubiquitously expressed under the control of the CAG promoter (Yahata, Yuasa, & Araki, 2009). Initially, we determined expression levels of Nmnat3 protein in Nmnat3 Tg and wild‐type (WT) mice. Nmnat3 levels were robustly increased in skeletal muscle, heart, and brain (Figure 1a). However, Nmnat3 contents in the liver were almost comparable between Nmnat3 Tg and WT mice (Figure 1a). Other tissues, including WAT and BAT, had moderate overexpression of Nmnat3 in Nmnat3 Tg mice (Figure 1a). Thus, we examined whether overexpression of Nmnat3 resulted in increased NAD levels in these tissues using Liquid chromatography–mass spectrometry (LC/MS). Consistent with protein levels of Nmnat3, NAD was significantly increased in skeletal muscle, heart, WAT, BAT, and brain, but not in liver of Nmnat3 Tg mice (Figure 1b). These results indicate that increased expression of Nmnat3 in mice efficiently boosts NAD levels in vivo.

### 2.2 NAD levels in Nmnat3 Tg mice were maintained after high‐fat diet (HFD) feeding or aging

Several studies have shown that obesity causes declines in skeletal muscle NAD levels (Yoshino et al., 2017). Thus, we investigated whether Nmnat3 overexpression can compensate for NAD deficiencies in skeletal muscle due to diet‐induced obesity. Initially, we confirmed that NAD levels were significantly decreased in skeletal muscle of HFD‐fed WT mice (Figure 1c). In HFD‐fed Nmnat3 Tg mice, NAD levels were also slightly decreased but were maintained at the same levels as in WT mice fed a normal chow diet (NC; Figure 1c). We also determined levels of the NAD precursor NAM in these mice and observed no changes due to HFD feeding in WT and Nmnat3 Tg mice (Supporting information Figure S1A). Because many studies have indicated that skeletal muscle NAD levels decline with aging (Frederick et al., 2016; Yaku, Okabe, & Nakagawa, 2018; Zhang et al., 2016), we determined NAD levels in WT and Nmnat3 Tg mice at the ages of 4, 18, and 24 months. Nmnat3 Tg mice had twofold higher NAD levels than WT mice at 3 months of age. Although NAD levels in WT mice decreased at 18 and 24 months of age, NAD levels in aged Nmnat3 Tg mice were similar to those in young mice (Figure 1d). In contrast to NAD, NAM levels were almost unchanged during aging (Supporting information Figure S1B), indicating that declines in NAD concentrations are not due to shortages of precursor. In addition, no significant differences in Nampt levels were observed between aged Nmnat3 Tg and WT mice (Supporting information Figure S1C).

### 2.3 Nmnat3 Tg mice have improved glucose tolerance after HFD feeding

**Figure 2:**
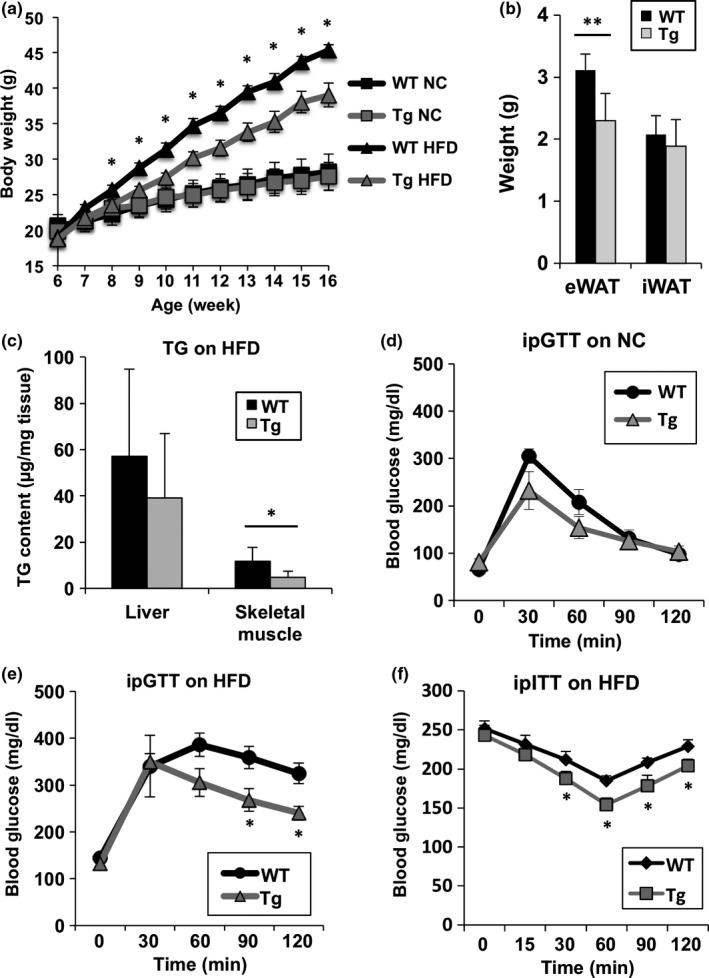
Nmnat3 Tg mice had improved glucose tolerance after HFD feeding. (a) Body weight changes of male WT and Nmnat3 Tg mice after NC or HFD feeding. Mice were fed the HFD after 6 weeks of age. Data are presented as mean ± *SD* (*n* = 12–16 for each group). (b) Adipose tissue weight after HFD feeding for 10 weeks. Data are presented as mean ± *SD* (*n* = 4 for each group). (c and d) Glucose concentrations in an intraperitoneal glucose tolerance test of WT and Nmnat3 Tg mice fed the NC (c) or HFD (d) for 8 weeks. Data are presented as mean ± *SD* (*n* = 6–8 for each group). (e) Glucose concentrations in an intraperitoneal insulin tolerance test of WT and Nmnat3 Tg mice fed the HFD for 8 weeks (*n* = 8 for each group). (f) TG contents in the liver and skeletal muscle from WT and Nmnat3 Tg mice fed the HFD for 10 weeks (*n* = 5–6 for each group). Single (*) asterisk indicates that *p*‐value was <0.05

To investigate whether increased NAD levels influence metabolism in vivo, we compared body weights of male WT and Nmnat3 Tg mice after feeding with a NC or HFD (Figure 2a). Although body weights were similar in these animals when fed a NC diet, HFD‐induced body weight gains were significantly less in Nmnat3 Tg mice than in WT mice (Figure 2a). We also evaluated food intake during NC and HFD feeding, and they were unchanged between Nmnat3 Tg and WT mice (Supporting information Figure S2). To quantify changes in adiposity after HFD feeding, we measured adipose tissue weights and found that Nmnat3 Tg mice gained less epididymal adipose tissue than WT mice (Figure 2b). We also determined triglyceride (TG) contents in the liver and skeletal muscle after HFD feeding. Although there was no significant difference in liver TG levels, we found that skeletal muscle of Nmnat3 Tg mice had less TG contents than those in WT mice (Figure 2c). Thus, we determined the impact of increased NAD levels on glucose metabolism using glucose tolerance tests (GTT) in Nmnat3 Tg and WT mice. Both Nmnat3 Tg and WT mice had almost normal glucose tolerance when fed the NC diet. Moreover, although WT mice had impaired glucose tolerance after HFD feeding, Nmnat3 Tg mice exhibited significantly improved glucose metabolism under the same conditions (Figure 2d and e). In subsequent investigations of insulin sensitivity in these mice using insulin tolerance tests (ITT), Nmnat3 Tg mice had better insulin sensitivity than WT mice following HFD feeding (Figure 2f). Taken together, these data indicate that HFD‐fed Nmnat3 Tg mice have superior glucose tolerance than HFD‐fed WT mice, reflecting improved insulin sensitivity.

### 2.4 Nmnat3 Tg mice have improved glucose tolerance during aging

**Figure 3:**
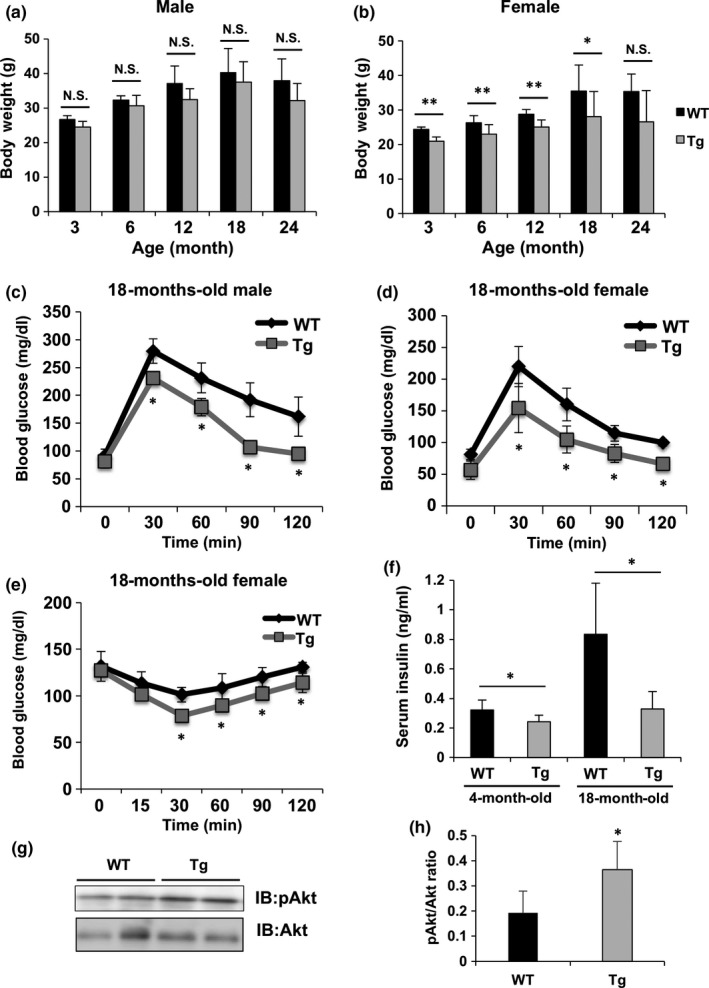
Aged Nmnat3 Tg mice had improved glucose tolerance. (a and b) Body weight changes of male (a) and female (b) WT and Nmnat3 Tg mice at the ages of 3, 6, 12, 18, and 24 months. Data are presented as mean ± *SD* (*n* = 10–16 for each group). (c and d) Glucose concentrations in an intraperitoneal glucose tolerance test of male (c) and female (d) WT and Nmnat3 Tg mice at the age of 18 months. Data are presented as mean ± *SD* (*n* = 8 for each group). (e) Glucose concentrations in an intraperitoneal insulin tolerance test of female WT and Nmnat3 Tg mice at the age of 18 months (*n* = 4 for each group). (f) Insulin concentrations in WT and Nmnat3 Tg mice at the ages of 4 and 18 months (*n* = 6–10 for each group). (g and h) Immunoblot analysis (g) of insulin‐stimulated Akt phosphorylation in the skeletal muscle from WT and Nmnat3 Tg mice at the age of 18 months. Quantification was performed by densitometry analysis (h) (*n* = 5–6 for each group). Single (*) and double (**) asterisk indicate that *p*‐value was <0.05 and 0.01, respectively

To assess the effects of increased NAD levels on aging and aging‐associated glucose intolerance in Nmnat3 Tg mice, Nmnat3 Tg and WT mice fed the NC diet were observed until 24 months of age. Body weights of female Nmnat3 Tg mice were significantly lower than those of control mice. However, no significant differences were observed between the body weights of male Nmnat3 Tg and WT mice (Figure 3a and b). To evaluate the glucose metabolism in aged Nmnat3 Tg mice, we performed GTT in 18‐month‐old Nmnat3 Tg and WT mice. In these tests, WT mice showed significantly impaired glucose tolerance with aging, whereas both male and female Nmnat3 Tg mice maintained almost normal glucose metabolism (Figure 3c and d). Consistent with the results from HFD‐fed Nmnat3 Tg mice, aged Nmnat3 Tg mice had improved insulin sensitivity in ITT (Figure 3e). Insulin concentrations in Nmnat3 Tg mice at 4 months of age were also lower than in age‐matched control mice (Figure 3f), and those in WT mice increased with aging, indicating the progression of insulin resistance in aging WT mice (Figure 3f). In contrast, Nmnat3 Tg mice retained lower insulin concentrations at 18 months of age, demonstrating that Nmnat3 Tg mice had better insulin sensitivity. In agreement, increased insulin‐stimulated Akt phosphorylation was observed in aged Nmnat3 Tg mice (Figure 3g and h). Taken together, these findings indicate that increased Nmnat3 expression alleviates aging‐associated glucose intolerance by increasing insulin sensitivity.

### 2.5 The SIRT1–PGC1α axis was not affected in Nmnat3 Tg mice

In previous reports, administration of NAD precursors, such as NMN and NR, increased NAD levels in various murine tissues (Canto et al., 2012; Yoshino et al., 2011). In these models, increased NAD levels activated the SIRT1–PGC1α axis and prevented the metabolic disorders that are associated with aging and obesity. Therefore, we determined whether Nmnat3‐mediated increases in NAD concentrations also activate the SIRT1–PGC1α axis. SIRT1 activates PGC1α through deacetylation and promotes the transcription of target genes involved in mitochondrial biogenesis. Thus, we initially determined expression levels of transcription factors that control genes of mitochondrial biogenesis. We found that TFAM and PGC1α expression levels declined after aging, but no significant differences between Nmnat3 Tg and WT mice were observed (Supporting information Figure S3A). We also assessed the acetylation status of PGC1α in aged Nmnat3 Tg mice but again found no significant changes in comparison with WT mice (Supporting information Figure S3B). Accordingly, PGC1α target genes were not upregulated in Nmnat3 Tg mice compared with those in WT mice, and these observations were similar in young and aged mice (Supporting information Figure S3C and D). These results suggest that Nmnat3‐mediated increases in NAD contents did not contribute to the activation of the SIRT1–PGC1α axis.

### 2.6 Mitochondrial NAD levels are increased in Nmnat3 Tg mice

**Figure 4:**
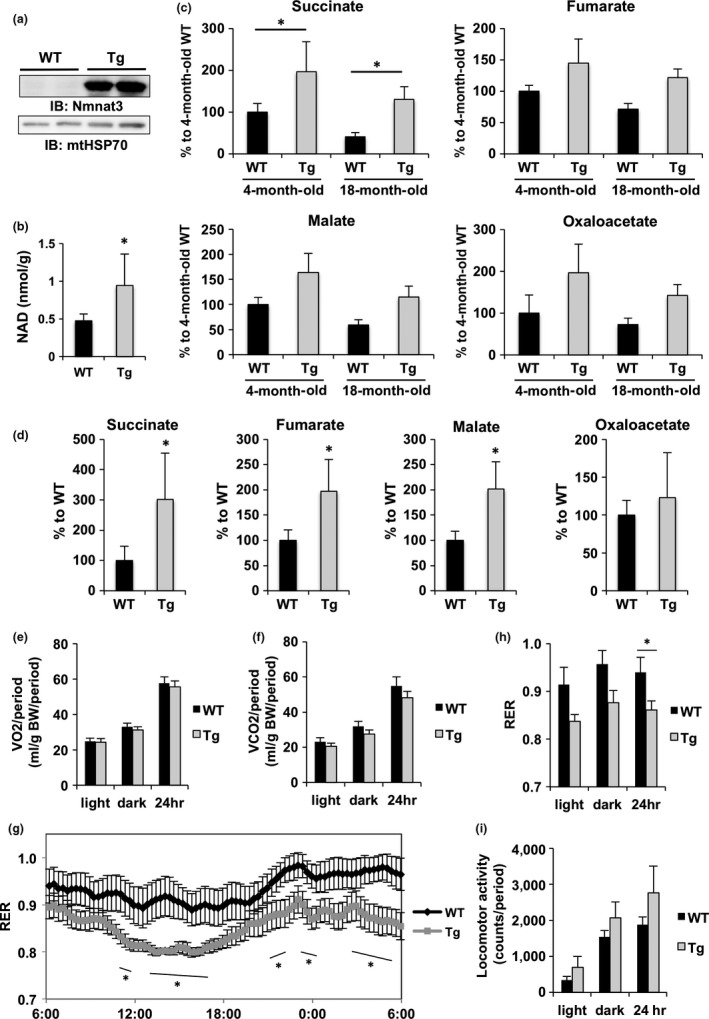
Mitochondrial NAD levels and metabolic pathways were enhanced in Nmnat3 Tg mice. (a) Immunoblot analysis of Nmnat3 expression in the mitochondria from skeletal muscle of 4‐month‐old female Nmnat3 Tg and wild‐type (WT) mice. mtHSP70 was used as loading control. (b) Absolute quantification of mitochondrial NAD levels in skeletal muscle. Tissue samples were prepared from female 4‐month‐old WT and Nmnat3 Tg mice. Data are presented as mean ± *SD* (*n* = 4 for each group). (c and d) Metabolites in the TCA cycle were measured by SIM mode‐operated GC/MS using skeletal muscle samples from the aged (c) or HFD‐fed (d) female WT and Nmnat3 Tg mice. The TCA cycle intermediates, including the fumarate, succinate, oxaloacetate, and malate, are measured by GC/MS. Data are presented as mean ± *SD* (*n* = 4 for each group). (e–h) Period summaries of oxygen consumption (VO_2_) (e), carbon dioxide production (VCO_2_) (f), and respiratory exchange ratio (RER) (g) were evaluated by indirect calorimetry using female 7‐month‐old Nmnat3 Tg and wild‐type (WT) mice (*n* = 8 for each group). Trend chart of RER is also displayed in (h). (i) Locomotor activity was evaluated using female 7‐month‐old Nmnat3 Tg and wild‐type (WT) mice (*n* = 8 for each group). Single (*) asterisk indicates that *p*‐value was <0.05

Previously, we demonstrated that loss of Nmnat3 does not influence mitochondrial NAD levels in mice. We also found that most endogenous Nmnat3 proteins reside in the cytoplasm (Hikosaka et al., 2014; Yamamoto et al., 2016), although several reports claim that Nmnat3 is present in the mitochondria (Nikiforov et al., 2011; VanLinden et al., 2015). However, we also reported that overexpression of Nmnat3 in HeLa cells encouraged localization of Nmnat3 in mitochondria (Hikosaka et al., 2014). Thus, we examined whether overexpression of Nmnat3 contributes to mitochondrial NAD levels in Nmnat3 Tg mice. In these experiments, endogenous Nmnat3 protein was not detectable in the mitochondria from skeletal muscles of WT mice, but considerable amounts of Nmnat3 were observed in skeletal muscle mitochondria from Nmnat3 Tg mice (Figure 4a). Consistent with this result, mitochondrial NAD levels were significantly increased in Nmnat3 Tg mice (Figure 4b), indicating that Nmnat3 overexpression boosts mitochondrial NAD levels in vivo.

### 2.7 TCA cycle and fatty acid oxidation were enhanced in Nmnat3 Tg mice

NAD controls the activity of various mitochondrial metabolic enzymes, including those of the TCA cycle, β‐oxidation, and oxidative phosphorylation (Scheibye‐Knudsen, Fang, Croteau, Wilson, & Bohr, 2015). Increases in mitochondrial biogenesis and function have previously been shown to improve insulin sensitivity (Bonnard et al., 2008; Joseph, Adhihetty, & Leeuwenburgh, 2016). Furthermore, caloric restriction (CR), which is currently the only nutritional intervention that delays aging and extends lifespan in mammals, is known to stimulate mitochondrial metabolism (Civitarese et al., 2007; Lopez‐Lluch et al., 2006). Thus, we investigated the impacts of increased mitochondrial NAD levels on energy metabolism in Nmnat3 Tg mice. Initially, we determined levels of TCA cycle metabolites using gas chromatography–mass spectrometry (GC/MS). The TCA cycle intermediates, succinate, fumarate, malate, and oxaloacetate, were present at significantly increased levels in skeletal muscles of young Nmnat3 Tg mice compared with those in WT mice (Figure 4c). Moreover, whereas the levels of these metabolites decreased with aging in WT mice, aged Nmnat3 Tg mice had similar metabolite levels to those in young Nmnat3 Tg mice (Figure 4c). We also investigated these TCA cycle metabolites in HFD‐fed mice and found that similar to the data from aging animals, Nmnat3 Tg mice had higher TCA cycle metabolite levels than WT mice (Figure 4d). Subsequently, we investigated oxygen consumption in Nmnat3 Tg and WT mice using indirect calorimetry. Although no significant differences in oxygen consumption (Figure 4e and Supporting information Figure S4A) or carbon dioxide production (Figure 4f and Supporting information Figure S4B) were observed between Nmnat3 Tg and WT mice, Nmnat3 Tg mice had lower respiratory exchange rates (Figure 4g and h), suggesting preferential utilization of fatty acid over carbohydrate as energy sources in Nmnat3 Tg mice. We also investigated locomotive activities in these mice and found no significant differences, although a tendency for higher locomotive activity levels was observed in Nmnat3 Tg mice (Figure 4i and Supporting information Figure S4C). These results indicate significant activation of energy metabolism pathways in the mitochondria of Nmnat3 Tg mice, even after aging.

### 2.8 Mitochondrial electron transport chain complex II, but not complex I, was present at increased levels in Nmnat3 Tg mice

**Figure 5:**
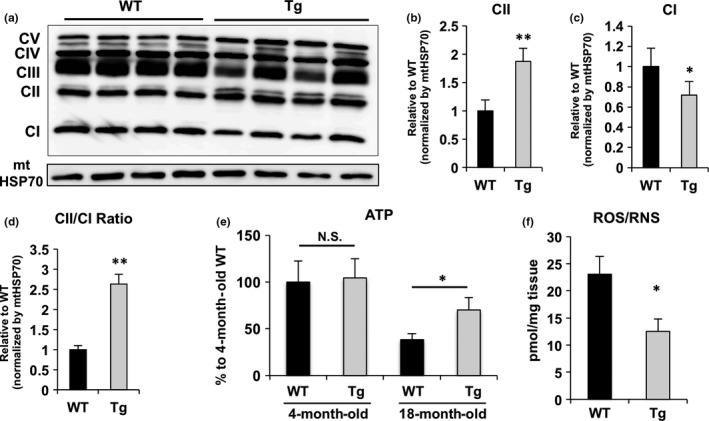
Nmnat3 Tg mice had the increased ATP and reduced ROS levels. (a) Immunoblot analysis of ETC. protein in skeletal muscle mitochondria from 7‐month‐old Nmnat3 Tg and wild‐type (WT) mice (*n* = 4 for each group). mtHSP70 was used as loading control. (b–d) Densitometry analysis was performed using ImageJ. The density values of complex I (b), complex II (c), and ratio of complex I to complex II (d) were normalized by mtHSP70. Data are presented as mean ± *SD* (*n* = 4 for each group). (e) Measurement of ATP levels in skeletal muscle samples from young (4‐month‐old) and old (18‐month‐old) female WT and Nmnat3 Tg mice. Data are presented as mean ± *SD* (*n* = 6 for each group) (f) Measurement of ROS levels in skeletal muscle samples from 18‐month‐old female WT and Nmnat3 Tg mice. Data are presented as mean ± *SD* (*n* = 5 for each group)

Mitochondria are energy centers that produce ATP but are also the primary source of ROS, which reportedly induce cellular damage in skeletal muscle and precede the onset of insulin resistance (Bonnard et al., 2008). In the mitochondria, ROS are mainly produced by electron leakage from complexes I and III (Murphy, 2009). Therefore, we investigated the effects of Nmnat3 overexpression on ATP and ROS production in skeletal muscle of Nmnat3 Tg mice. Initially, we evaluated expression levels of mitochondrial electron transport chain (ETC.) proteins using western blotting with an anti‐ETC. antibody cocktail. These analyses showed that among the five mitochondrial ETC. protein complexes, complex II expression was significantly increased (Figure 5a and b). Conversely, complex I protein levels were remarkably decreased in Nmnat3 Tg mitochondria (Figure 5a and c), leading to significantly higher ratios of complex II to complex I in Nmnat3 Tg mice (Figure 5d). It has been reported that mitochondria under CR have increased complex II activity and generate less ROS from complex I (Aspnes et al., 1997). In agreement, we have found that complex II substrates, such as succinate and fumarate, were significantly increased in Nmnat3 Tg mice (Figure 4c). Thus, we speculated that mitochondria of Nmnat3 Tg mice generate ATP more efficiently and produce less ROS. To confirm these assertions, we measured ATP levels in skeletal muscle using LC/MS and found that ATP levels declined with aging in WT mice, but were maintained in Nmnat3 Tg mice, even after aging (Figure 5e). Conversely, ROS production was markedly diminished in aged Nmnat3 Tg mice (Figure 5f), suggesting that increased NAD levels in Nmnat3 Tg mice improve the efficiency of mitochondrial respiration, presumably through increased activities of complex II.

### 2.9 Levels of NGD were dramatically increased in Nmnat3 Tg mice

**Figure 6:**
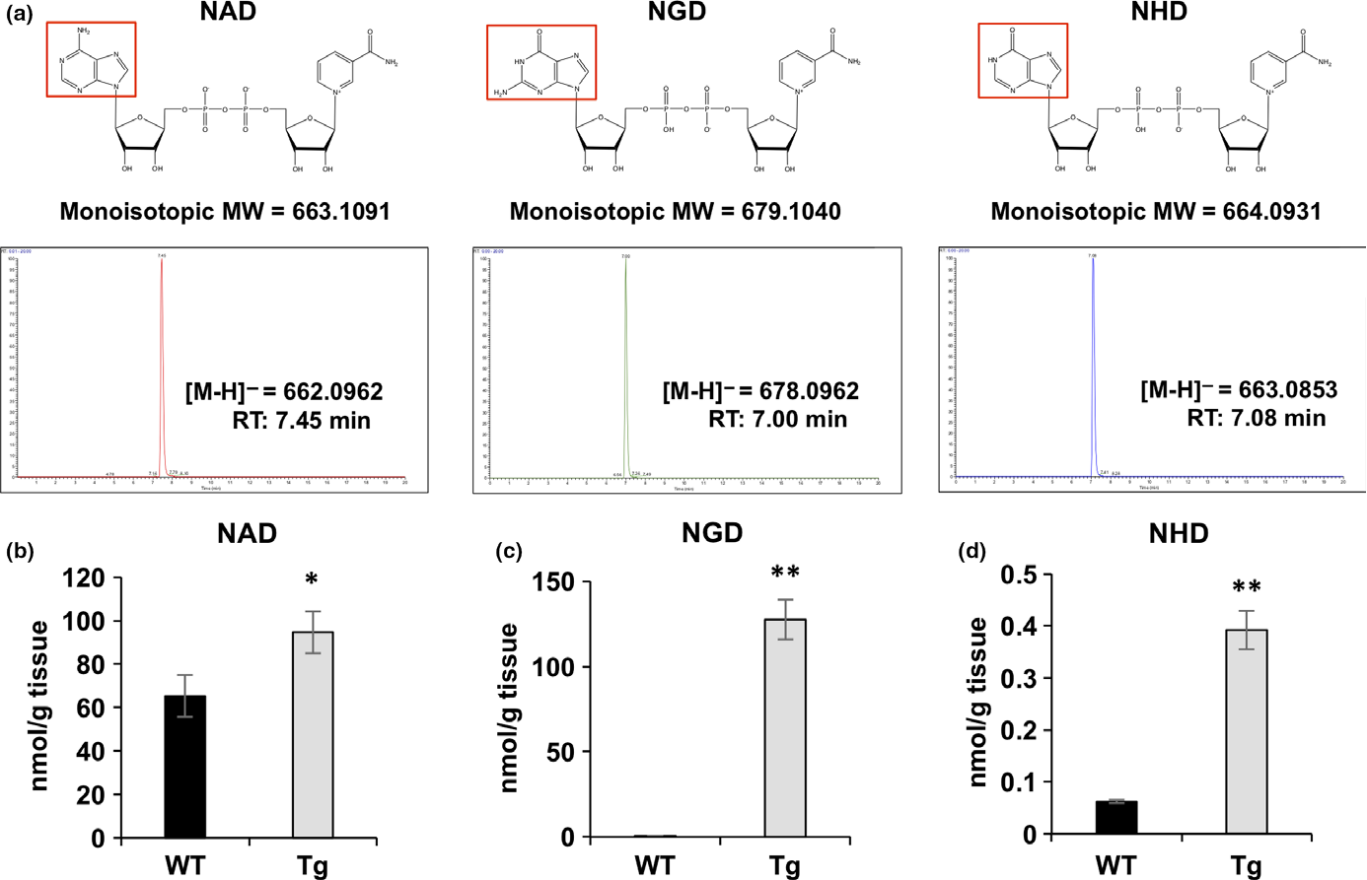
Overexpression of Nmnat3 significantly increased NGD and NHD levels in vivo. (a) Chemical structures and representative chromatogram of standard NAD, NGD, and NHD. 10 pmol standard solution was injected into the FT‐MS (LTQ Orbitrap XL). (b–d) Absolute quantification of NAD (b), NGD (c), and NHD (d) levels using skeletal muscle tissue samples prepared from WT and Nmnat3 Tg mice. Data are presented as mean ± *SD* (*n* = 4 for each group)

Nmnat3 reportedly produces the NAD analogs NGD and NHD from GTP and ITP, respectively (Berger et al., 2005). However, these findings were only obtained using in vitro studies with recombinant proteins, and the presence of endogenous NGD and NHD in vivo is yet to be verified. To address this issue, we determined whether NGD and NHD levels are increased in Nmnat3 Tg mice by developing a method for specifically detecting NGD and NHD using Fourier transform mass spectrometry (Orbitrap) with high‐performance liquid chromatography. In these experiments, we distinguishably detected NAD, NGD, and NHD using standard compounds (Figure 6a) and then applied the method to tissue samples from WT and Nmnat3 Tg mice. Although endogenous NGD and NHD were detectable in skeletal muscle tissues from WT mice, these metabolites were present at very low concentrations in comparison with NAD (Figure 6b–d). However, NGD levels were dramatically increased in Nmnat3 Tg mice (Figure 6c). We also observed significant increases in NHD levels in Nmnat3 Tg mice, whereas absolute NHD concentrations were much lower than those of NAD and NGD (Figure 6d). Because NGD levels were almost comparable to those of NAD in Nmnat3 Tg mice, we speculated that NGD plays functional roles in the mitochondria. NGD has similar chemical structures to NAD, suggesting that NGD could competitively inhibit complex I. To verify this possibility, we examined inhibitory effects of NGD to complex I in vitro and showed that NGD inhibited complex I mildly but significantly (Supporting information Figure S5), potentially leading to changes in mitochondrial function in Nmnat3 Tg mice. However, it remains unclear whether increased NGD and NHD levels actually contributed to the phenotypes of Nmnat3 Tg mice, and further studies are warranted to decipher these roles in vivo.

## 3 DISCUSSION

Aging is a highly complex physiological process and is influenced by both genetic and environmental factors. Many studies have indicated that increased free radical levels contribute significantly to aging (Harman, 2003). In particular, mitochondria are a major source of free radicals including ROS, and it is accepted that mitochondrial dysfunction contributes considerably to the aging process (Bratic & Larsson, 2013; Murphy, 2009). It has also been shown that CR increases numbers of healthy mitochondria and promotes ATP production in rodents and humans (Civitarese et al., 2007). In addition, CR led to reduced oxygen consumption in mice, but efficient ATP production was maintained with decreased ROS generation (Guarente, 2008). In the present study, aged Nmnat3 Tg mice maintained ATP production and generated less ROS than their aged WT counterparts. Excessive intracellular ROS in skeletal muscles reportedly induces cellular damage and precedes the onset of insulin resistance (Bonnard et al., 2008). Therefore, reduced ROS levels would likely be protective against diet‐induced and aging‐associated insulin resistance in Nmnat3 Tg mice. In accordance with the roles of NAD as a coenzyme in mitochondrial fatty acid oxidation, we observed the preferential use of fatty acids as an energy source in Nmnat3 Tg mice, and such shifts from carbohydrate‐ to fatty acid‐based energy production have been considered metabolically favorable during aging (Nguyen, Samson, Reddy, Gonzalez, & Sekhar, 2013; Schonfeld, Wieckowski, Lebiedzinska, & Wojtczak, 2010; Tucker & Turcotte, 2002). In addition, reduced capacity for fatty acid oxidation is related to insulin resistance (Kelley, Goodpaster, Wing, & Simoneau, 1999). Thus, increased NAD may activate fatty acid oxidation and likely contribute to the maintenance of insulin sensitivity during aging (Supporting information Figure S6).

### 3.1 Possible mechanisms of reduced ROS levels in Nmnat3 Tg mice

In our experiments, mitochondria from Nmnat3 Tg mice had increased levels of complex II and decreased levels of complex I (Figure 5a–d). Complex I oxidizes NADH and transfers an electron to ubiquinone (UQ), whereas complex II uses succinate as a substrate and transfers an electron to UQ (Murphy, 2009). Complex II activities and protein expression levels were shown to be decreased during aging in a number of studies (Aspnes et al., 1997; Bowman & Birch‐Machin, 2016; Wojtovich, Smith, Haynes, Nehrke, & Brookes, 2013). Moreover, several reports demonstrate that reverse electron transfer (RET) from complex II to complex I triggers ROS production in the mitochondria (Batandier et al., 2006; Liu, Fiskum, & Schubert, 2002; Schonfeld et al., 2010), and increased ROS during aging or HFD feeding was largely attributable to RET from complex II to complex I (Brand, 2010; Vial et al., 2015). Therefore, increased ratios of complex II to complex I in Nmnat3 Tg mice probably reduce ROS generation and ameliorate age‐associated insulin resistance. However, the regulatory mechanism of these ETC. proteins in Nmnat3 Tg mice has not been characterized and should be the focus of future investigations.

### 3.2 NAD–SIRT1–PGC1α axis in Nmnat3 Tg mice

Previously, dietary interventions with the NAD precursors NMN and NR have been shown to increase NAD levels and ameliorate diet‐induced obesity and diabetes (Canto et al., 2012; Yoshino et al., 2017). In these models, increased NAD levels induced deacetylation of PGC1α and subsequently stimulated mitochondrial biogenesis and activity. However, although NAD levels in Nmnat3 Tg mice were increased by two‐ to threefold compared with those in WT control mice, the SIRT1–PGC1α axis was not activated in our experiments (Supporting information Figure S3). Hence, Nmnat3 overexpression may instead increase mitochondrial NAD levels and promote efficient mitochondrial respiration, resulting in high ATP production and low ROS generation. From this perspective, Nmnat3 is an ideal target that may directly boost mitochondrial NAD levels and stimulate mitochondrial functions.

### 3.3 Biological functions of NGD and NHD

To our knowledge, this is the first study to identify endogenous NGD and NHD in vivo, and our experiments show that overexpression of Nmnat3 significantly increases the presence of these NAD analogs in skeletal muscle. In addition, we showed that NGD inhibits complex I activity in vitro (Supporting information Figure S5). We speculate that under these conditions, NGD mimics NAD and competitively inhibits NADH oxidizing reactions. However, the contributions of increased NGD and NHD to the phenotypes in Nmnat3 Tg mice remain unclear. Similar to NAD, plasma and mitochondrial inner membranes may be impermeable to NGD and NHD. Thus, it may be difficult to investigate their functions by directly administrating these NAD analogs to isolated mitochondria, cells, or mice. Furthermore, their effects may be indistinguishable from those of NAD. Because all of these NAD analogs are generated from NMN, administration of NAD precursors or activation of Nampt possibly increases the levels of all NAD analogs, depending on Nmnat isozyme abundance or the availability of ATP analogs. Thus, it is of interest to engineer NGD‐ or NHD‐directed Nmnat3 mutants and examine their functions in mice to distinguish the biological roles of NGD and NHD from NAD.

### 3.4 Limitations of the study

To investigate the role of Nmnat3 during aging, ubiquitously Nmnat3‐overexpressed mice were used in this study. The ensuing Nmnat3 protein levels were increased in most tissues except for the liver and efficiently led to increased NAD levels and improved metabolic health during aging. However, Nmnat3 overexpression levels in these mice were dramatic in some tissues, including in skeletal muscle, potentially undermining the relevance of these observations to physiological conditions, in which Nmnat3 levels are increased by certain stimuli. Our experiments also showed that moderate overexpression of Nmnat3 in WAT and BAT was sufficient to increase NAD levels in these tissues. Thus, it may be important to determine Nmnat3 expression levels that optimally increase mitochondrial NAD levels in vivo. In addition to NAD synthesis activity, Nmnat3 possesses reported chaperone‐like activities that may be beneficial independently of NAD production (Zhai et al., 2008). Finally, in this study, we only demonstrated the benefits of Nmnat3 overexpression in skeletal muscle, and several studies have shown roles of NAD and sirtuins in the regulation of insulin sensitivity in other tissues, including adipose tissues and brain (Chalkiadaki & Guarente, 2012; Sasaki et al., 2014; Satoh et al., 2013; Stromsdorfer et al., 2016; Yoon et al., 2015). We also observed significant increases in NAD levels in WAT, BAT, and brain. Thus, insulin sensitivity may also be regulated by increased NAD levels through sirtuin‐dependent and sirtuin‐independent mechanisms in these Nmnat3 Tg mouse tissues. Thus, future investigations using tissue‐specific Nmnat3‐overexpressing mouse models are warranted to clarify the roles of overexpressed Nmnat3 in adipose tissues and brain.

In summary, our study demonstrates that Nmnat3 gain of function efficiently increases NAD and NGD concentrations, with potential metabolic benefits during aging. Taken together, the present data warrant further consideration of Nmnat3 as a promising target for the treatment of various age‐associated metabolic diseases.

## 4 EXPERIMENTAL PROCEDURES

### 4.1 Mice

Nmnat3 mice were generated as described previously (Yahata et al., 2009). Ectopic Nmnat3 was expressed ubiquitously under the control of CAG promoter. The mice were maintained under a standard light cycle (12‐hr light/dark) and were allowed free access to water and food. For high‐fat‐induced obesity experiments, mice were fed high‐fat diet containing 60% fat (Research Diets). All of the animal care policies and procedures for the experiments were approved by the animal experiment committee at the University of Toyama.

### 4.2 Western blot analysis

Whole‐tissue lysates were prepared from WT and Nmnat3 Tg mice. Mice were sacrificed and tissues for western blot were immediately frozen in liquid nitrogen and preserved in −80°C until utilization. Harvested whole tissues were ground by Multibeads shocker (Yasui Kikai) with RIPA buffer (500 mM NaCl, 1% Nonidet P‐40, 0.5% sodium deoxycholate, 0.1% SDS, 50 mM Tris‐HCl, pH 7.4) and then subjected to western blot analysis. WAT and BAT were ground by Multibeads shocker (Yasui Kikai) with lysis buffer (10 mM Tris‐HCl, 2 mM EDTA, 0.1% Nonidet P‐40, 150 mM NaCl). Lysates were centrifuged to remove insoluble materials. Lysates were mixed with sample buffer before protein denaturation with boiling at 95°C for 5 min. Protein lysates were run in 7.5% separating gel and transferred afterward to PVDF Immobilon‐P transfer membrane (Millipore, Billerica MA). Membrane was incubated overnight at 4°C with the primary antibody and for 1 hr at room temperature with the secondary antibody. Images were detected by LAS 4000 Mini digital imager (GE Healthcare). Quantification of immunoblot bands was performed by the densitometry using ImageJ software (NIH). Primary antibodies used for western blot were anti‐mouse Nmnat3 rat monoclonal antibody (originally produced), total OXPHOS Rodent WB Antibody Cocktail (Abcam), anti‐acetyl‐lysine antibody (CST), anti‐Akt (CST), p‐Akt (CST), anti‐GAPDH (Sigma), anti‐β‐actin (Wako), and anti‐mtHSP70 (Abcam). For immunoprecipitation, anti‐lPGC1α (Santa Cruz Biotechnology) was used.

### 4.3 Metabolite extraction for LC/MS and GC/MS

Whole tissues were ground using Multibeads shocker (Yasui Kikai) with LC/MS‐grade methanol and water in equal proportion by volume and then centrifuges at 13,000 g for 5 min at 4°C. Supernatant was mixed with an equal volume of chloroform. The upper aqueous phase was dried using Speedvac SPD 1010 (Thermo). For LC/MS experiments, the dried pellet of sample was dissolved in 60 μl LC/MS‐grade water (Wako) and then passed through a filter with 0.45‐μm Millex filter unit (Millipore). For GC/MS experiments, the derivatization of samples was carried out in two steps. In the first step, carbonyl functional groups were protected by methoximation using 20 μl of 20 mg/ml solution of methoxyamine hydrochloride in pyridine at 30°C for 90 min. In the second step, after adding 80 μl N‐methyl‐N‐trimethylsilyltrifluoroacetamide with 1% trimethylchlorosilane (MSTFA + 1% TMCS; Pierce), samples were incubated at 37°C for 30 min for derivatization.

### 4.4 Metabolite measurement for LC/MS and GC/MS

The levels of NAD and NAD‐related metabolites including ATP were determined by multiple reaction monitoring (MRM) using Agilent 6460 Triple Quad mass spectrometer coupled to Agilent 1290 HPLC system. MS settings and chromatographic conditions were used as described previously (Yaku et al., 2018). TCA cycle intermediates were analyzed by selected ion monitoring (SIM) using Agilent 5977 MSD Single Quad mass spectrometer coupled to Agilent 7890 Gas Chromatography. A 30‐cm‐long DB5‐MS column with 10‐m DuraGuard precolumn was used for separation by GC/MS. Helium was used as a gas carrier with a constant flow rate of 1.1 mL/min. The temperature setting was started at 60°C for 1 min, then increased at 10°C/min to 325°C, and held at 325°C for 10 min. Amounts of metabolite were calculated by integrated sum of area using MassHunter Quantitative Software (Agilent).

### 4.5 Measurement of NGD and NHD

Standard compounds of NGD and NHD were purchased from Sigma (USA). Measurements of these NAD analogs were performed using LTQ Orbitrap XL mass spectrometer coupled with HPLC (Thermo Scientific). We employed the FT scan mode with negative ESI and acquired the data with rigorous mass point to less than four digits. NAD analogs were separated on Waters Atlantis T3 column (2.1 × 150 mm, 3 μm) with 10 μl volume injection and at a flow rate of 150 μl/min using 5 mM ammonium formate for mobile phase A and 100% methanol for mobile phase B. The setting for chromatographic gradients was as follows: 0–10 min, 0–70% B; 10–15 min, 70% B; and 15–20 min, 0% B. The data were extracted by mass range between the rigorous mass of NAD analogs up to plus and minus 0.01 using Xcalibur software (Thermo).

### 4.6 Indirect calorimetry and locomotor activity measurement

Oxygen consumption and CO_2_ production were measured in individual mice at the indicated age using an Oxymax apparatus (Columbus Instruments, Columbus, OH, USA). The O_2_ and CO_2_ measurements were performed every 18 min for each mouse over a 3‐day period, and the data from the final day were analyzed. Locomotor activity was measured with the ACTIMO‐100 (Shinfactory, Fukuoka, Japan) (Sasaki et al., 2014, 2015).

### 4.7 Statistical analysis

Analysis was performed using an unpaired or paired Student's *t* test and one‐way ANOVA with the post hoc Tukey test. Data are expressed as mean ± *SD*, and significant differences are confirmed statistically when *p*‐value is <0.05.

## CONFLICT OF INTEREST

None of the authors have any conflict of interests.

## AUTHOR CONTRIBUTIONS

TN and KT conceived and designed the experiments. MG, KY, KO, AM, TS, and TN performed the experiments and analyzed the data. MY, KH, TK, and IU contributed to reagents/materials/analysis tools. GM, KY, KO, and TN wrote the manuscript. All authors reviewed the manuscript.
