# Supplementary material for: Overexpression of Nmnat3 efficiently increases NAD and NGD levels and ameliorates age‐associated insulin resistance
Source: Aging Cell. 2018 Jun 14;17(4):e12798. doi: 10.1111/acel.12798 (PMC6052485; doi:10.1111/acel.12798)
Supplement: Supplementary file 1 [file ACEL-17-na-s001.pdf]

Supplementary Information

Overexpression of Nmnat3 efficiently increases NAD and NGD levels and ameliorates age-associated insulin resistance

Maryam Gulshan, Keisuke Yaku, Keisuke Okabe, Arshad Mahmood, Tsutomu Sasaki, Masashi Yamamoto, Keisuke Hikosaka, Isao Usui, Tadahiro Kitamura, Kazuyuki Tobe, and Takashi Nakagawa

Experiment Procedures

Isolation of mitochondria

Isolation of mitochondria from mouse tissues was described elsewhere. In brief, skeletal muscles were excised from hind limb, and were homogenized in Buffer MA (67mM Sucrose, 50mM Tris-HCl pH7.4, 50mM KCl and 10mM EDTA), followed by the centrifugation by the same scheme described above. The pellet was dissolved in Buffer MB (250mM Sucrose, 10mM Tris-HCl pH7.4 and 3mM EGTA) and used as mitochondria.

Real-time quantitative PCR

Total RNAs were extracted from mouse tissues using TRI Reagent (Molecular Research Center, Inc.). cDNA was prepared using ReverTra Ace qPCR RT Master Mix with gDNA Remover (Toyobo, Japan) according to the supplier's protocol. Real-time PCR was carried out using THUNDERBIRD SYBR qPCR Mix (Toyobo) on Thermal Cycler Dice Real Time System II (Takara Bio). Quantification was done by Delta Delta Ct method, and *Rpl13a* gene was used as a reference gene.

A glucose tolerance test (GTT) and an insulin tolerance test (ITT)

For the GTT experiments, the mice were fasted for 16 h and then injected with glucose (1g/Kg body weight) intraperitoneally. For the ITT experiments, the mice were fasted for 4 h, and then intraperitoneally injected with human insulin (0.8U/Kg body weight and 0.3U/Kg for HFD and normal chow fed mice, respectively). The blood glucose concentration was measured using an automatic blood glucose meter (NOVA Biomedical). The serum insulin levels were determined by the Mouse Insulin ELISA KIT (AKRIN-031; Shibayagi).

ROS measurement

Amount of ROS was determined using Oxiselect^{TM} In Vitro ROS/RNS Assay Kit (Cell Biolabs Inc.) according to supplier's manual. Tissue lysate were grinded by Multibeads shocker (Yasui Kikai) in PBS with 1% NP40 and 0.1% SDS. For measurement, samples were diluted to ten times with PBS, and ROS amount was calculated using hydrogen peroxide standard curve.

Complex I activity assay

The activity of complex I was measured using MitoCheck® Complex I Activity Assay Kit (Cayman) according to the manufacture's instruction. In this experiment, bovine heart mitochondria were incubated with NADH in the presence or absence of 1mM NGD or 1μM rotenone. The absorbance of NADH (340 nm) was monitored using Varioskan multi plate reader (Thermo). The slope was calculated from linear portion of plotted absorbance. The activity was represented as relative to the value of slope in the control (absence of inhibitor).

Supplementary Figure 1

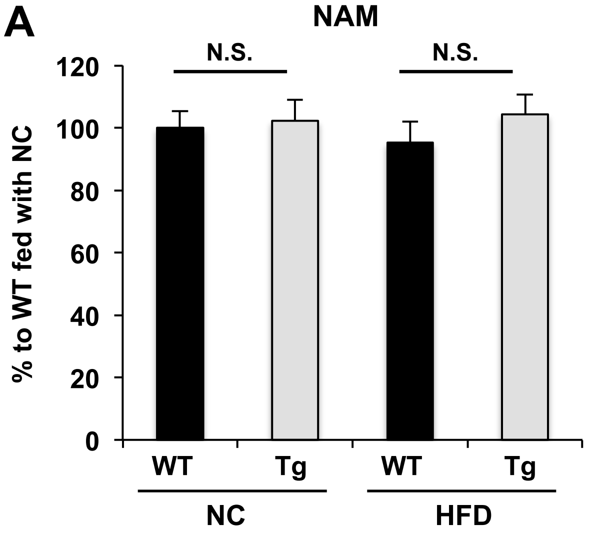

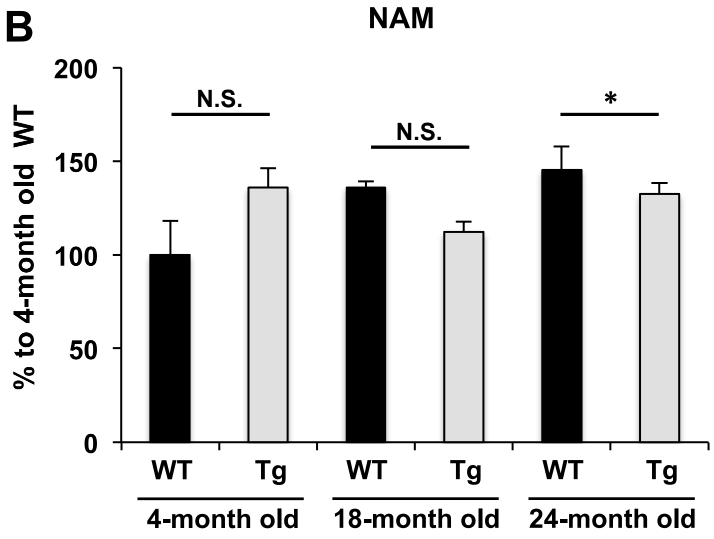

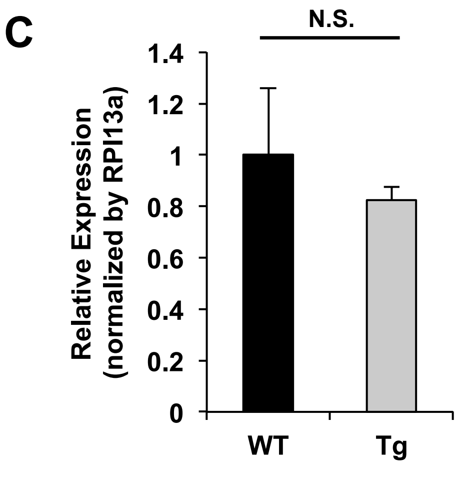

Supplementary Figure 2

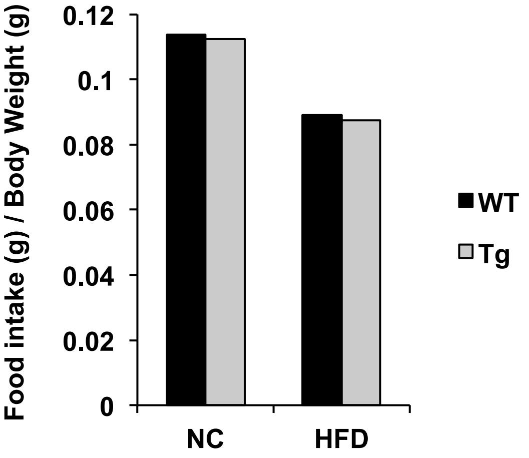

Supplementary Figure 3

A

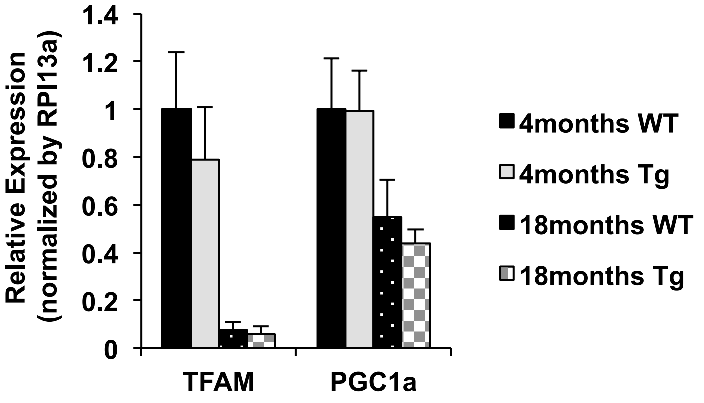

B

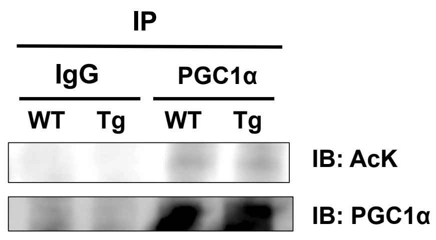

C

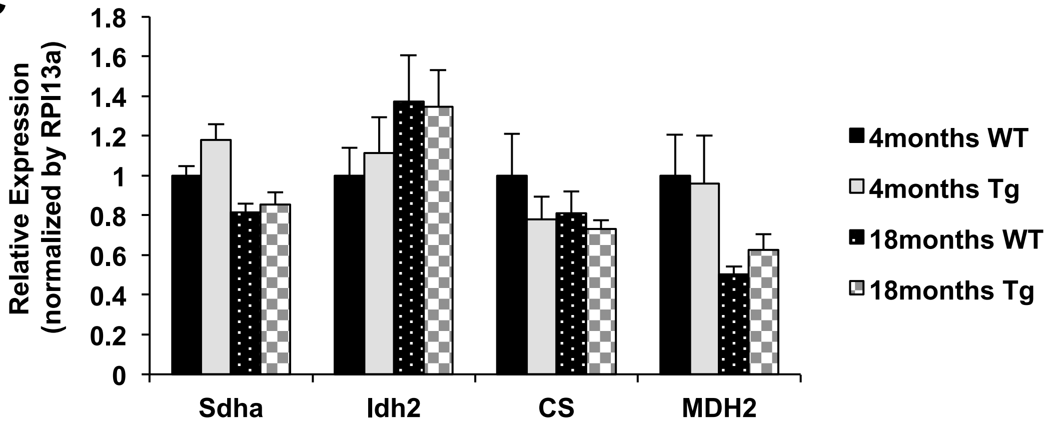

D

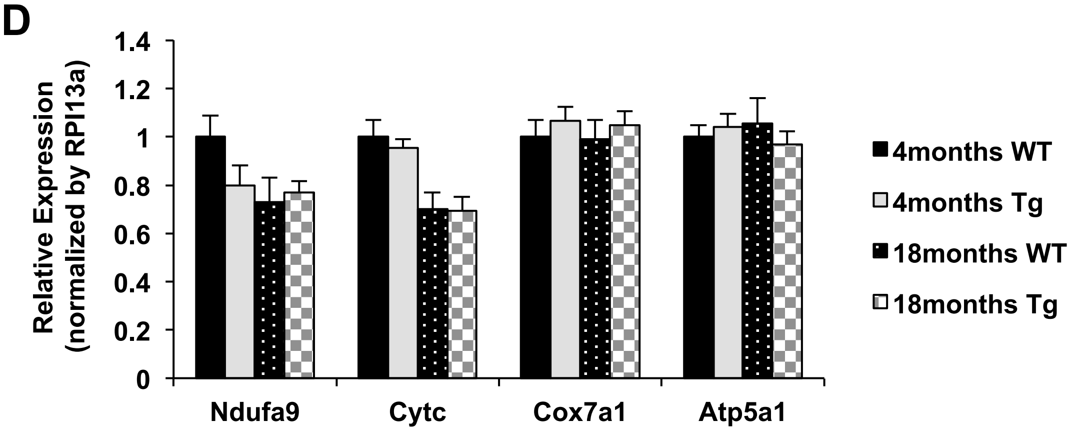

Supplementary Figure 4

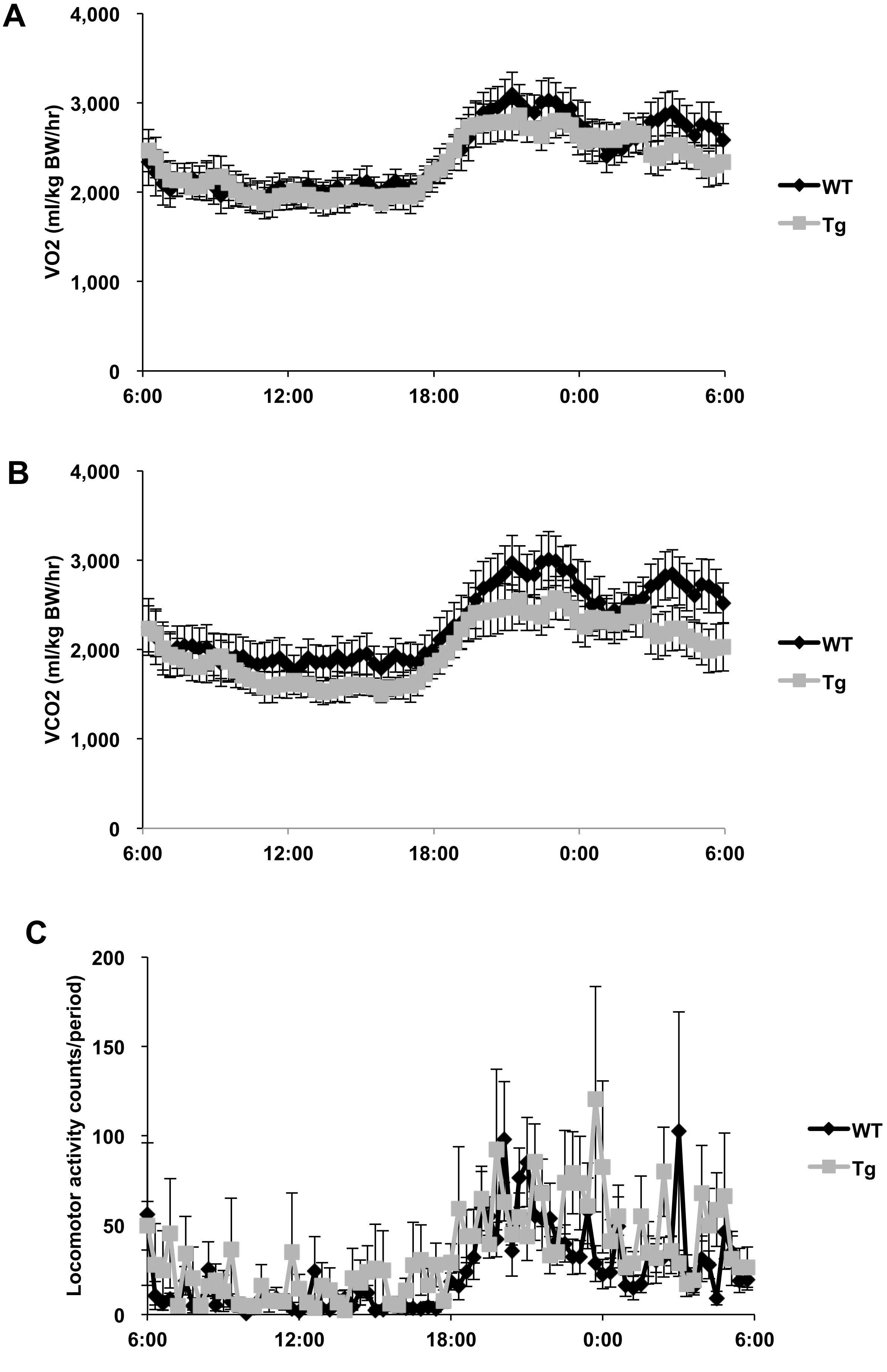

Supplementary Figure 5

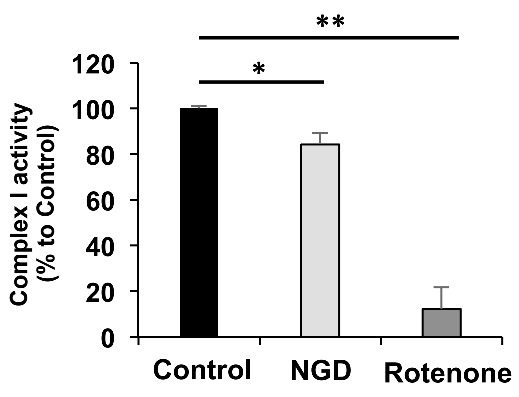

Supplementary Figure 6

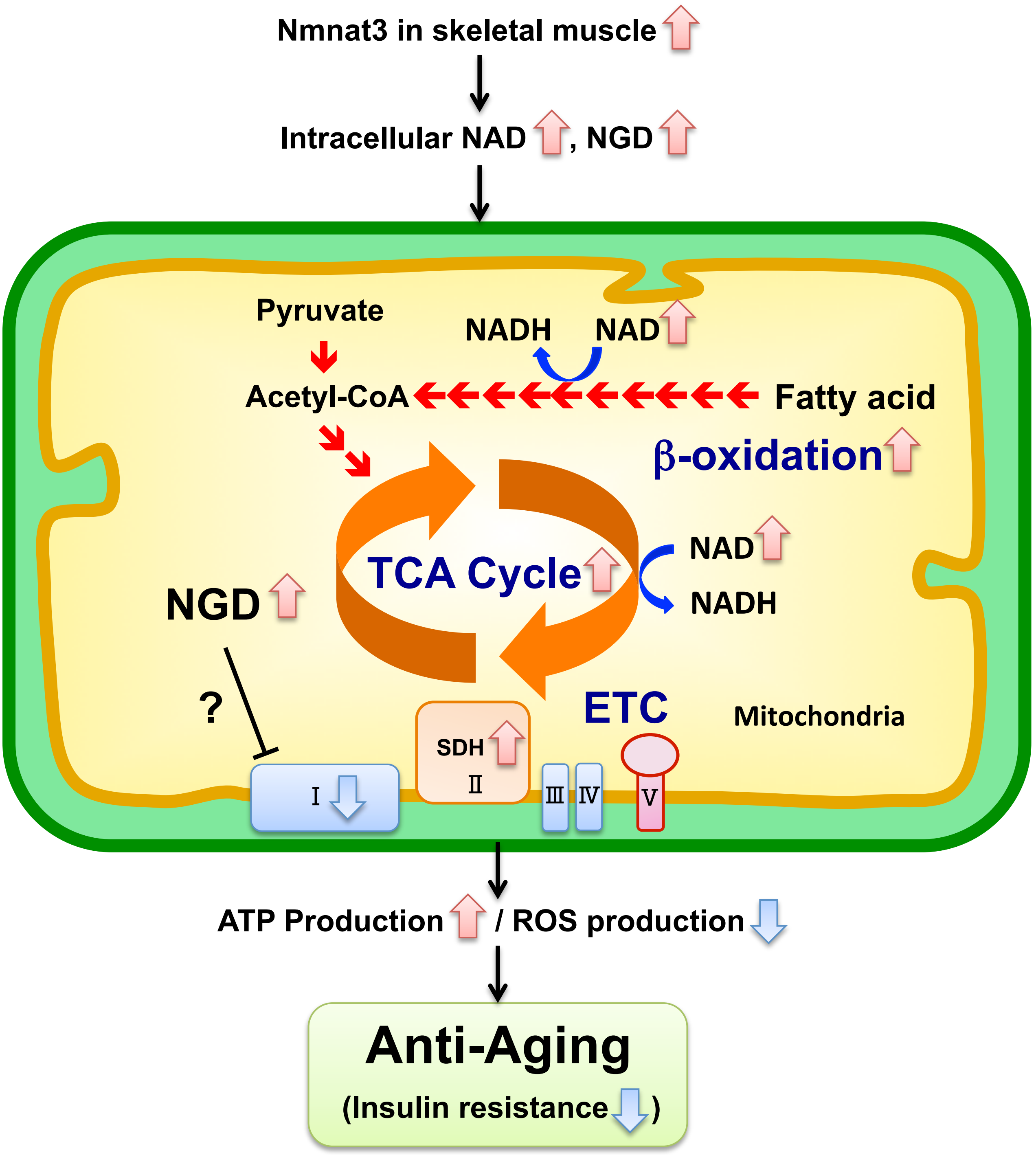

Supplemental Figures Legends

Supplemental Figure 1

(A) Semi-quantification of NAM levels by LC/MS using tissue samples prepared from female WT and Nmnat3 Tg mice fed with NC or HFD for 10 weeks. Data are presented as mean ± SD (n=8 for each group). (B) Semi-quantification of NAM levels by LC/MS using issue samples were prepared from female WT and Nmnat3 Tg mice at the age of 4-, 18, 24-month old. Data are presented as mean ± SD (n=8 for each group). (C) Real-time quantitative PCR analysis for mRNA level of Nampt. Total RNA was isolated from skeletal muscle of WT and Nmnat3 Tg mice. *Rpl13a* gene was used as a reference gene, and data are presented as a relative value to WT for each gene (n=5 for each group).

Supplemental Figure 2

Food intake was calculated from 7-week old WT and Nmnat3 Tg mice fed on NC or HFD. Values were average of four mice in each group.

Supplemental Figure 3

(A) Real-time quantitative PCR analysis for mRNA level of mitochondrial biogenesis genes including TFAM and PGC1α. (B) Immunoprecipitation using anti-PGC1α antibody and subsequent western blotting with anti-acetyl-Lysine antibody. Samples were prepared from the skeletal muscle of WT and Nmnat3 Tg mice. (C and D) Real-time quantitative PCR analysis for mRNA level ofmitochondrial enzyme genes including Sdha, Idh2, Cs, Mdh2, (B) and mitochondrial ETC genes including Ndufa9, Cytc, Cox7a1 and Atp5a1 (C). Total RNA was isolated from skeletal muscle of WT and Nmnat3 Tg mice. *Rpl13a* gene was used as a reference gene, and data are presented as a relative value to WT for each gene (n=8 for each group).

Supplemental Figure 4

(A and B) Trend chart of oxygen consumption (A) and carbon dioxide production (VCO_2) (B) was represented. Data were evaluated using 7-month old female Nmnat3 Tg and wild-type (WT) mice (n=8 for each group). (C) Trend chart of

locomotor activity was represented. Data were evaluated using 7-month old female Nmnat3 Tg and wild-type (WT) mice (n=8 for each group).

Supplemental Figure 5

In virto complex I activity assay using bovine heart mitochondria. 1 mM NGD or 1 μM rotenone, a well-known complex I inhibitor, was used for this experiment. Data were obtained from three independent experiments, and presented as mean ± SD. Single (*) and double (**) asterisk indicated that *p*-value was less than 0.05 and 0.01, respectively.

Supplemental Figure 6.

Gain-of-function Nmnat3 exhibited the metabolically beneficial effects *in vivo*. Schematic of the proposed protection mechanism against aging-associated insulin resistance in Nmnat3 Tg mice.
